# Characterization of Hemagglutinin Antigens on Influenza Virus and within Vaccines Using Electron Microscopy

**DOI:** 10.3390/vaccines6020031

**Published:** 2018-05-25

**Authors:** John R. Gallagher, Dustin M. McCraw, Udana Torian, Neetu M. Gulati, Mallory L. Myers, Michael T. Conlon, Audray K. Harris

**Affiliations:** Laboratory of Infectious Diseases, National Institute of Allergy and Infectious Diseases, National Institutes of Health, 50 South Drive, Room 6351, Bethesda, MD 20892, USA; john.gallagher2@nih.gov (J.R.G.); dustin.mccraw@nih.gov (D.M.M.); utorian@niaid.nih.gov (U.T.); neetu.gulati@nih.gov (N.M.G.); mallory.myers@nih.gov (M.L.M.); michael.conlon@nih.gov (M.T.C.)

**Keywords:** influenza, vaccines, structure, electron microscopy, cryo-EM, design

## Abstract

Influenza viruses affect millions of people worldwide on an annual basis. Although vaccines are available, influenza still causes significant human mortality and morbidity. Vaccines target the major influenza surface glycoprotein hemagglutinin (HA). However, circulating HA subtypes undergo continual variation in their dominant epitopes, requiring vaccines to be updated annually. A goal of next-generation influenza vaccine research is to produce broader protective immunity against the different types, subtypes, and strains of influenza viruses. One emerging strategy is to focus the immune response away from variable epitopes, and instead target the conserved stem region of HA. To increase the display and immunogenicity of the HA stem, nanoparticles are being developed to display epitopes in a controlled spatial arrangement to improve immunogenicity and elicit protective immune responses. Engineering of these nanoparticles requires structure-guided design to optimize the fidelity and valency of antigen presentation. Here, we review electron microscopy applied to study the 3D structures of influenza viruses and different vaccine antigens. Structure-guided information from electron microscopy should be integrated into pipelines for the development of both more efficacious seasonal and universal influenza vaccine antigens. The lessons learned from influenza vaccine electron microscopic research could aid in the development of novel vaccines for other pathogens.

## 1. Introduction

**Figure 1:**
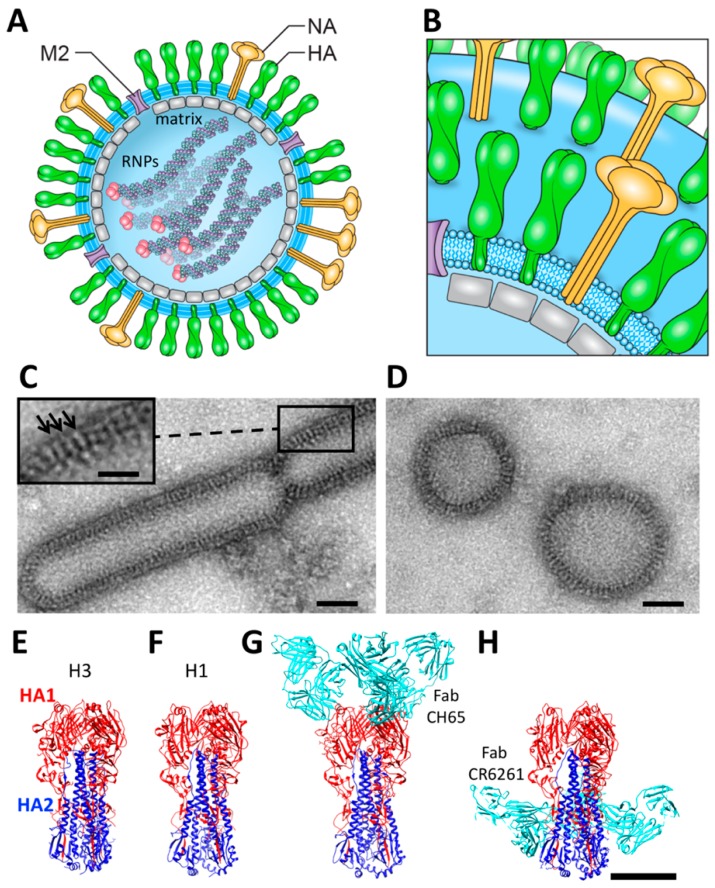
Influenza virus organization and hemagglutinin (HA) structure. (**A**) schematic of an influenza virus particle. Viral glycoproteins are hemagglutinin (HA, green); neuraminidase (NA, yellow); matrix 2 (M2) (purple). The membrane is shown in light blue. Genomic ribonucleoprotein complexes (RNP) filaments are inside with a trimeric viral polymerase complex (pink) at the end of each RNP; (**B**) the most populous glycoprotein on the virion surface is HA, which is accompanied by lesser amounts of NA, and a minority of M2; (**C**,**D**) influenza virus particles negatively stained with PTA; (**C**) Influenza virus, A/Victoria/3/75 (H3N2), displaying filamentous morphology. Scale bar, 50 nm; (**C**, inset) individual glycoprotein spikes on the virion surface are indicated with arrows. Scale bar, 25 nm; (**D**) A/Victoria/3/75 virions with spherical morphologies. Scale bar, 50 nm; (**E**–**H**) trimeric HA ectodomains solved by protein X-ray crystallography. HA1 is shown in red and HA2 is blue with Fabs in cyan. (**E**) H3 HA (PDB 4O5N); (**F**) H1 HA (PDB 3LZG); (**G**) H1 HA in complex with Fab CH65 bound to receptor binding site (PDBID 5UGY); and (**H**) H1 HA in complex with Fab CR6261 bound to the stem region (PDB 3GBN). Scale bar, 5 nm. Panel images are originals created for this review.

Influenza virus is an enveloped virus composed of several structural layers that lack defined radial symmetry (Figure 1A). The segmented, single-stranded RNA genome is found at the center of the viral particle and is complexed with viral nucleoprotein to form ribonucleoprotein complexes (RNPs) [1,2,3]. The viral envelope that surrounds the RNPs consists of an inner layer of matrix, formed by the M1 protein. Viral surface glycoproteins hemagglutinin (HA), neuraminidase (NA) and matrix 2 (M2) span the viral membrane (Figure 1B). HA and NA project from the viral surface and can be recognized by neutralizing antibodies [4,5,6,7]. The ability of HA antibodies to agglutinate virus particles and neutralize the virus has led to HA being formulated as the major antigen in current commercial influenza vaccines. HA continues to be a major focus in structure-guided efforts to design HA immunogens to display more conserved epitopes. These epitopes include the conserved receptor binding site and the stem region of HA [8,9,10,11,12,13,14,15,16,17]. Cryo-electron microscopy (cryo-EM) has contributed to the knowledge of other important components of influenza viruses, such as size, shape, and RNP arrangements in spherical and elongated viruses [18,19]. In addition, cryo-EM has played a role in understanding the matrix layer and fusion events [20,21,22,23,24]. However, we will focus on HA studies related to the structural analyses of HA in various contexts such as ectodomains, viruses, and vaccines.

It is well established that antibodies to HA can reduce morbidity and mortality associated with influenza infection [25]. This has led to HA comprising the main antigen in influenza vaccines [26]. There are different types of influenza vaccines including live-attenuated influenza viruses [27,28,29,30,31,32], inactivated split-subunit vaccines [33,34,35,36] and recombinant HA [37,38,39]. Existing influenza vaccines need to be reformulated annually to address antigenic changes in HA. In total, influenza type A viruses are divided into antigenically distinct subtypes of HA (H1–H18) [40]. Influenza type B viruses also circulate in humans and are divided into two lineages: Yamagata and Victoria [41]. Current vaccine designs focus on HA antigens currently circulating in humans using multivalent formulations, including H1 and H3 for influenza A, and an HA from Yamagata and/or Victoria lineage viruses for influenza B [42].

The goal for future influenza vaccines is to provide protection against both influenza type A viruses of varied subtypes, as well as type B viruses of both lineages. This is often termed as the development of a “universal influenza vaccine”. A number of strategic meetings on universal influenza vaccines have taken place and more are planned. For example, in order to better define future objectives and goals for pathways leading to more efficacious seasonal and universal influenza vaccines, the National Institute of Allergy and Infectious Diseases (NIAID) conducted a meeting to gauge the state of influenza vaccine research and to get input from investigators from academia and industry. This resulted in a strategic plan for a universal influenza vaccine [43,44]. Defined objectives included expanding the understanding of influenza transmission, identifying targets for improved disease control measures, identifying alternative mechanisms of protection beyond HAI-mediating antibodies, supporting rational design of universal influenza vaccines via design of new immunogens that elicit a wider breadth of protection, and providing research resources and tools to improve influenza vaccines [43]. We think that research driving the understanding of the structural biology of influenza virus is contributing important information that will aid in accomplishing shared objectives in developing a universal influenza vaccine.

In this review, we will highlight structural biology work related to influenza virus research mainly using electron microcopy (EM). HA presentation has been studied by EM to reveal the structures and epitope display for virus particles, influenza subunits vaccines, and designed immunogens for nanoparticle vaccines [4,10,11,16,39,45,46]. From numerous and diverse studies, a key contribution of EM to influenza vaccine design is to relate the structure and accessibility of HA in vaccine candidates to that on the virus. EM is uniquely capable of visualizing the mode of antibody binding to native antigen. Here, we focus on HA-based vaccines because HA is immunodominant among influenza epitopes, and HA is the current target of influenza vaccine design. Interest in other influenza glycoproteins is growing rapidly and NA and M2 each show great promise, and are reviewed elsewhere [47,48,49]. Future studies will allow a continuum of structural biology research. This will improve the design of conserved epitopes as presented on vaccine immunogens in order to match similar epitopes of infecting viruses, as well as aid in improving anti-viral responses by the immune system to conserved epitopes for more efficacious vaccines.

## 2. Structure of Influenza Virions and Surface Spikes by Electron Microscopy

The structure of influenza glycoproteins and their organization on the virion surface is important because these spikes (e.g., HA and NA) are the target of neutralizing antibodies [50,51]. Whether presenting these antigens with different spacing and arrangements could promote or hinder their immunogenicity is still an active area of investigation. Subunit vaccines and virus-like particles are being developed to increase accessibility of particular epitopes, with the goal of boosting their immunogenicity. On a larger scale, morphology of the entire virion can vary in length from 100 nm to 1 μm, which may have a role in influenza transmission [52], and electron microscopy remains the primary means of differentiating spherical from filamentous morphology virions.

Although influenza vaccines were introduced around the 1940s [33], understanding the 3D molecular structures of influenza virus and their epitopes followed decades later [4,8,13,45,53,54]. Over the years, these structural techniques have evolved with innovations in automated data collection strategies, improved detectors, and experiments conducted at liquid nitrogen temperatures (i.e., cryo). In particular, low temperature aids to capture native-state structural information for hydrated biological samples. These structural methods continue to inform on the location and interaction between antibodies and epitopes on influenza antigens, such as HA and NA [8,9,12,13,16,45,53,55,56,57,58,59].

Some of the first structural views of influenza virus particles and surface glycoprotein spikes were revealed by electron microscopy using the negative staining technique [6,7,60]. This technique involves adhering particles to a thin carbon surface before staining with heavy metal and dehydration. The result is strong image contrast, which can often reveal the molecular shapes of macromolecules down to approximately 15 Å resolution [61]. Although the negative staining technique was introduced by Brenner and Horne in 1959 [61], it is still used today to image virus samples (Figure 1C,D). Phosphotungstic acid (PTA) is a widely used negative stain for influenza virus that produces a good contrast for viral glycoprotein spikes at neutral pH. Other negative stains used to visualize influenza include methylamine tungstate (NanoW) [62] and uranyl acetate [63]. Depending on the application, the low pH of uranyl acetate and related uranium-free stains may be of concern because conformational change of HA is triggered by low pH. The mechanism of how negative stain is applied to the sample can have a profound impact on the resulting particle morphology, but this can be minimized with techniques such as “optimized negative staining” [64].

Electron microscopy of a negatively stained influenza virus identified the influenza virion as a non-symmetrical particle [6,7,60]. Influenza virions have different sizes and are referred to as pleiomorphic (Figure 1C,D). The overall virus morphology affects the curvature of the virus surface, which can impact epitope accessibility on influenza glycoproteins.

Influenza virus morphology can be categorized as spherical or filamentous. The influenza virus is also commonly found as a very short filament, which can be referred to as a capsule-shaped, or a bacilliform species. The different morphologies (Figure 1C,D) could impact antibody accessibility and reduce neutralizing antibody effectiveness of certain neutralizing antibodies. Electron microscopy has been used to determine that filamentous particles are more populous in primary isolates, while egg-adapted viruses used in vaccine production are more spherical [65]. This is important because one notion is that filamentous virions are more transmissible than spherical virions. The role of virus morphology in influenza transmission and infection is an area of active research. Interestingly, the matrix gene has been linked to filamentous morphology and transmission [66,67,68]. Because electron microscopy has shown to be uniquely suited to study the morphologies of influenza viruses and how adaption and mutations might change the morphology and the nature of transmission, more of this type of research is likely to continue in the future. This would aid in assessing if next-generation immunogens and vaccines are displaying conserved epitopes in a manner likely to elicit antibodies that will target the same epitopes on transmitting influenza viruses.

Interestingly, recombinant vaccines based upon heterologous expression systems of HA to form virus-like particles (VLPs) have been explored as a means to recapitulate the antigenic glycoprotein coating of influenza virus, while avoiding essential components for infectivity, such as the viral genome. VLPs have been expressed and validated by EM when produced from such systems as insect cells [69], as well as plant-based production systems [70,71]. Recombinantly produced VLPs offer the advantage over traditional influenza vaccine production in that HA and NA subtypes can be intermixed in any desired combination and immediately proceed into production. Additionally, VLPs have been tested in human clinical trials and have been shown to be safe and immunogenic [72,73].

## 3. Hemagglutinin Structures and Epitopes by X-Ray Crystallography

Four decades after the introduction of experimental influenza vaccines, protein X-ray crystallography was used to solve the first structure of an influenza HA ectodomain in the early 1980s [33,53]. The first crystal structure was of an H3 HA, and it established a protein architecture now prototypical of hemagglutinins (Figure 1E–H) [53,74]. The HA ectodomain is trimeric, and the constituent HA polypeptide chains can be viewed as being folded into head and stem domains. An alternative nomenclature widely used for the stem is the stalk. Head and stem domains are created from proteolytic cleavage of a single polypeptide (HA0). This cleavage is essential for function and has been implicated as a mechanism behind increased pathogenesis during bacterial coinfection and by H5 strains that are naturally highly-pathogenic [75]. The head domain (HA1) is a globular domain made up of mostly beta strands (Figure 1E, HA1 (red)). The stem domain (HA2) is composed mostly of alpha helices (Figure 1E, HA2 (blue)). At low pH, HA changes conformation to mediate virus entry, resulting in a presumed dissociation of the HA1 domains, and a refolding of the HA2 domain into a longer and narrower stalk [76]. We focus primarily on the pre-fusion HA structure because it is the relevant species for vaccine design.

There are hundreds of HA ectodomain structures solved by X-ray crystallography in the Protein Data Bank (PDB). The HA structure is conserved among the different HA subtypes (H1–H18), as illustrated by the structures of H1 and H3 subtypes (Figure 1E,F) [74,77]. By aligning the sequences of H1 and H3, the sequence identity between HA1 domains is 35%, and the sequence identity between HA2 domains is 52%, yet the structures are highly similar (Figure 1E,F). Additional sequence divergence in HA head domains is due to selective pressure during infection. Mutations in the head domain have been used to elucidate HA epitopes and antigenic sites in the head region of HA [53,78,79,80]. The head domain does have a conserved role in receptor binding, and X-ray crystallography has given insights into the localization of the receptor binding site for sialic acid and how antibodies target this site. The receptor binding site is located at the top of the HA1 domain, and broadly neutralizing antibodies, such as CH65, target this conserved site (Figure 1G) [12,13,81,82]. Many epitopes have been identified by X-ray crystallography, mapping important antigenic sites and illustrating how antibodies can target conserved regions in the HA stem regions [8,9,17,57,83]. One example of a broadly reactive stem antibody is CR6261 (Figure 1H) [8]. A number of investigators are now using information from both X-ray crystallography and electron microscopy in a complementary fashion to understand antigen structure and epitope organization.

## 4. Influenza Glycoproteins on Virions by Cryo-EM

Although X-ray crystallography has established the structure of soluble trimeric HA ectodomains (Figure 1E–H), influenza virions themselves are pleiomorphic and are refractory to crystallization. Thus, insights into how HA molecules are arranged in 3D on virions and how HA molecules on virions are bound by antibodies were elucidated by cryo-EM studies [4,16,45]. Cryo-EM is a technique in which samples in aqueous buffer are frozen more rapidly than ice crystals can form, resulting in vitreous ice [63,84,85]. Samples prepared in this manner are advantageous compared to negative staining because there is no chemical treatment of the sample—they are literally frozen in the native state [63,84,85].

**Figure 2:**
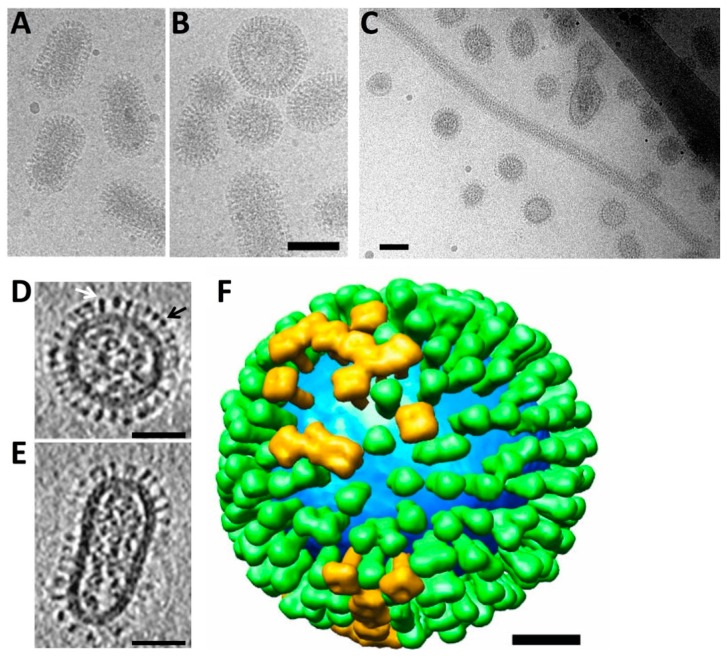
Influenza virions imaged by cryo-electron microscopy (cryo-EM). (**A**) capsule shaped influenza virions. Scale same as (**B**); (**B**) spherical and capsule shaped influenza virions. 100 nm scale bar; (**C**) a filamentous virus particle several microns in length among smaller spherical virions. 100 nm scale bar. (**D**,**E**) slices through 3D maps (i.e., tomograms) of a spherical virion (panel **D**) and a capsule-like virion (panel **E**). Arrows in panel (**D**) indicate an HA molecule with an apparent peanut shape (white arrow) and an NA molecule with an apparent mushroom shape (black arrow). Scale bars, 50 nm; (**F**) model of the surface distribution of HA (green) and NA (gold) on an influenza virus particle with membrane shown in blue. Scale bar, 20 nm. Virus analyzed is influenza X-31 H3N2. Panels (**A**–**C**) are originals and panels (**D**–**F**) were adapted from Harris et al. [4].

**Figure 3:**
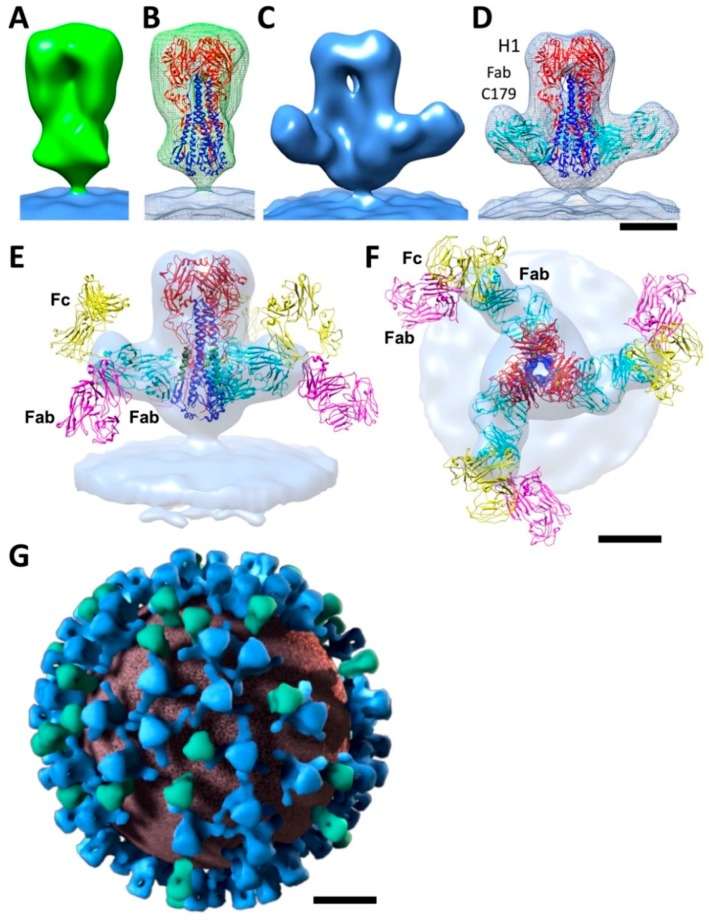
Three-dimensional structures of HA embedded in the viral membrane by cryo-electron tomography (cryo-ET). (**A**) side view of the 3D structure of H1 HA embedded in the viral membrane derived from cryo-electron tomography of 2009 H1N1 pandemic influenza virus. The 3D map is shown as a solid isosurface (green) with the membrane region colored blue; (**B**) docking of HA1 (red) and HA2 (blue) ectodomain coordinates (PDB 3LZG) into the density map of trimeric HA, shown as wire mesh; (**C**) side view of the density map of the complex formed by C179 antibody with trimeric HA. The HA-C179 map is represented as a solid isosurface (blue); (**D**) molecular model of the H1 HA-C179 complex by docking H1 HA (PDB 3LZG) and C179 Fab coordinates (PDB 4HLZ) into the HA-C179 map (wire-mesh); (**E**,**F**) top and side views, respectively, for a model of IgG molecules bound to C179 stem epitopes on viral HA. H1 coordinates (PDB 3LZG) were docked into the HA-C179 density map along with three surrogate IgG molecules (PDB 1IGY) based on Fab density protruding from the HA-C179 map. HA and Fabs are shown as ribbons, with HA1 in red, HA2 in blue, helix A of HA2 in green, the Fc region of the antibody in yellow. The non-bound Fab arm is magenta, and the Fab arm used to dock the IgG into the map in cyan. Scale bars, 5 nm; (**G**) 3D model of the distribution of unbound HA (green) and C179-bound HA (blue) spikes on a virus surface. Membrane is displayed as brick red. Scale bar, 20 nm. Virus is pandemic 2009 H1N1 influenza virus. Panels (**A**–**G**) were based on and adapted from Harris et al. [45]. Panels (**A**–**D**) used deposited 3D maps from the Electron Microscopy Data Bank (EMDB) (H1, EMD-5682; H1-C179, EMD-5684).

There are two major imaging modalities in electron microscopy as applied to biological samples imaged under cryo conditions. There is 2D cryo-EM in which 2D images are collected (Figure 2A–C). Commonly called single particle analysis, 2D cryo-EM can be used to take large numbers of 2D micrographs, which can be used to reconstruct a 3D volume when each 2D image is assumed to represent a different view of the average 3D particle structure. Alternatively, there is cryo-electron tomography (cryo-ET) in which a series of 2D images are collected at different angular tilts of the specimen, but on the same region of interest. This series of tilt images is used to computationally reconstruct a 3D volume or 3D map called a tomogram, which contains the entire field of view reconstructed in 3D. A key point is that cryo-ET determines a 3D structure from individual particles, and 3D structures of individual virus particles can be visualized (Figure 2D–F) [4]. Further details about glycoprotein conformations and their complexes with antibodies on the surfaces of viruses can be elucidated by using subtomogram averaging (Figure 3) [16,45].

Cryo-EM of the influenza virus indicates pleiomorphic structures that can be capsule-shaped (Figure 2A), spherical (Figure 2B), or filamentous particles that can be several microns in length (Figure 2C). Cryo-EM is suited to this type of research because cryo-EM can reveal the relative proportions of spherical to filamentous virions without artifacts from negative staining that may deform virus particles. Additionally, cryo-EM permits higher resolution details to be resolved. In cryo-EM of the influenza virus, spherical, capsule-shaped, and filamentous particles appear densely covered with viral glycoprotein spikes (e.g., HA). This suggests that morphological differences between influenza strains do not substantially affect glycoprotein surface density. Thus, it will be interesting in future vaccine studies using cryo-EM techniques to see how different viral subtypes, strains, and mutations affect glycoprotein distribution and conformation, and how this relates to epitope display and immunogenicity.

The conformation and 3D distribution of HA and NA molecules on the surfaces of influenza virions is one major question uniquely addressed by determining structures of influenza viruses by cryo-ET. The first 3D structures of spherical and capsule-shaped influenza virions imaged in 3D by cryo-ET were reported by Harris and colleagues [4], finding that some surface glycoproteins were present in clusters rather than randomly distributed on the virion surface. Cryo-ET revealed the overall 3D organization of influenza viruses with viral glycoproteins on the surfaces and genomic RNPs in the interior (Figure 2D,E). HA proteins can be identified within tomograms as peanut-shaped density, a hallmark of the HA prefusion conformation (Figure 2D, white arrow) while NA can be identified as mushroom-shaped density (Figure 2D, black arrow). This is consistent with shapes from the crystal structures of HA and NA ectodomains [4,45]. The spatial organization of longer filamentous viruses showed that NA clusters were often found at one end of the filament, while RNP clusters inside the virion were present at the opposite end [54]. From virus tomograms, a major finding was that the average distance between neighboring HA molecules was 14 nm [45], which posed a key question of whether HA stem epitopes are accessible on the virion surface. Despite the virion surfaces being densely covered with glycoprotein spikes, no ordered lattice interactions were observed, suggesting that variability in spike distances could accommodate bivalent antibody binding. This is illustrated by the distribution of HA and NA on a virion surface (Figure 2F). NA molecules were clustered, but not in a lattice (Figure 2F, gold). Additionally, these structural studies suggested that HA and NA epitopes could be equivalently exposed to antibody binding (Figure 2F).

## 5. Antibody Access to Stem Epitopes on Viruses by Cryo-ET

Cryo-ET can determine the spatial availability of conserved epitopes on the virion surface, and provides a direct means to test the availability of newly investigated epitopes targeted by new vaccine designs. For example, the packing density of influenza glycoproteins on the virion surface raised a question within the influenza vaccine field as to whether stem antibodies would be able to access their epitopes, or whether their binding would be sterically hindered. If indeed the HA stem was not accessible to antibodies on the virion surface, then the utility of vaccine-induced antibodies that bind the HA stem would be questionable, despite the presence of conserved epitopes in that location.

To address the question of stem epitope accessibility, cryo-ET was performed with the 2009 H1N1 pandemic influenza virus alone, and in complex with the stem antibody C179 [45]. To improve resolution of 3D volumes of individual glycoprotein spikes from tomograms (Figure 2D,E), volumes can be computationally extracted, aligned, and averaged. This is called subtomogram averaging and had been used for HIV [86,87]. The advantage of this strategy was that the HA 3D conformation could be observed as natively found on the surface of influenza viruses. Understanding the conformation of HA, i.e., whether it is in a pre- or post-fusion state, is important because epitopes for broadly-neutralizing stem antibodies, such as CR6261 (Figure 1H), have epitopes present only on the prefusion state of HA [8]. The 3D map of HA derived from tomograms had a peanut-shaped contour (Figure 3A). An overlay of HA ectodomain coordinates from X-ray crystallography and the cryo-ET subtomogram average map of virus HA was consistent with a pre-fusion state of HA (Figure 3B). Stem antibodies can block the conversion of HA from a pre-fusion to a post-fusion state required for virus entry [8,50,88]. The analysis was repeated in the presence of C179, and the structure of C179 bound to HA on the virus surface was determined by cryo-ET with subtomogram averaging. The resulting density maps indicated the structure of HA in the pre-fusion state with C179 density positioned at the stem region of HA (Figure 3C,D).

Although purified C179 IgG was used, only Fab shaped densities were observed in the 3D map of the HA-C179 complex (Figure 3C). However, molecular modeling using H1 and C179 coordinates docked into the map revealed a similar structure of viral H1-C179 to that of ectodomain H1-CR6261 complex from crystallography (Figure 3D vs. Figure 1H). This agreement was important because, in protein crystals, Fabs often make crystal contacts and their relative orientations could be different than Fab arms of IgGs on viral HA and affect the position of other Fab and Fc regions.

Numerous influenza antibodies are thought to exert anti-viral functions through their Fc regions via Fc receptors [89]. Fc-mediated immune responses can differ based on the modification of the Fc region and the particular Fc receptor [89,90,91,92,93]. To further understand how intact IgG with Fab and Fc arms would engage stem epitopes of viral HA, molecular modeling was used with IgG molecules modeled into the H1-C179 map (Figure 3E,F) [45]. Modeling suggested that both non-bound Fab and Fc arms may be flexible with the Fc region pointing away for the viral surface (Figure 3E,F). Fc orientation could be important for stem antibody function and this could be a structural basis for stem antibodies mediating Fc-related antibody activities, such as antibody-dependent cell-mediated cytotoxicity (ADCC) [89,90,91]. To address the HA stem accessibility to IgG molecules, computational sorting was performed to separate out HA that was bound by IgG C179 from unbound HA. The positions of these molecules were then mapped on the surface of viruses. An example is shown in Figure 3G [45]. The averaged maps for unliganded and liganded HA were used to denote the distribution. Approximately 70% of the HA molecules were bound by C179. It was found that HA and HA-C179 complexes were randomly distributed over the surface (Figure 3G). This suggested that each HA stem region had the equal chance of being bound by a C179 antibody. Thus, the density of viral glycoprotein spikes on the virion does not cause steric hindrance for the binding of antibodies to the majority of stem regions of HA molecules (Figure 3G). This key finding indicates that broadly-neutralizing stem-binding antibodies would have sufficient conformational space to bind epitopes at the side of HA, rather than the top. Similar cryo-ET studies indicated that influenza viruses displaying H1, H5 and chimeric H1/H5 HA molecules on their surfaces were bound by antibodies targeting both head and stem epitopes on HA [16]. Taken together, these cryo-EM studies on influenza viruses indicate the utility of direct observation of intact antibodies complexed with HA molecules on the surface of virions, and how these results may have important ramifications for further understanding Fc effector function.

Additionally, the Fc region of antibodies may play an important role in B-cell affinity, as indicated by Wang and co-workers. They reported that influenza vaccine efficacy is determined by B cell affinity selection driven by Fc–Fc receptor interaction affected by Fc glycans [93]. Whether such Fc antibody regions also help mediate immune response to different types of influenza vaccine immunogens needs to be studied in further detail. Future structural and biochemical studies on influenza subunit and nanoparticle vaccines with IgG molecules will be important in this regard. Previous work has established a basis for the use of cryo-EM in studying influenza subunit vaccines and designed nanoparticles as discussed below.

## 6. Molecular Organization of Hemagglutinin in Subunit Vaccines

HA is the major antigen in commercial influenza vaccines and is one of the main antigens being engineered to produce novel immunogens based on structure-guided design principles [10,11,14,15,94,95]. Some of the first viral glycoproteins studied by negative staining EM were isolated hemagglutinin complexes [96]. However, the organization of HA complexes from commercial influenza vaccines and the 3D structure of HA within the vaccines has not been studied in detail, illustrating gaps in our understanding of the organization and biochemistry of influenza vaccines. Could structural and biochemical differences of commercial vaccines affect the memory B-cell response and susceptibility of different age groups to potentially pandemic virus strains [97,98,99,100]? What can we learn from the organization of commercial influenza vaccines that informs structure-guided design of novel immunogens?

The biochemistry, organization and conformation of HA is important in determining what epitopes are recognized by the immune system and how vaccine-elicited antibodies target viral antigens. Vaccine epitopes could be variable, conserved, or misfolded and irrelevant to native HA. However, traditional subunit influenza vaccines with full-length HA have not been studied in structural detail because they have proven difficult to crystallize for protein X-ray crystallography, unlike crystallizable HA ectodomains (Figure 1E–H). These structurally heterogeneous samples are uniquely suited for structural analysis by EM.

**Figure 4:**
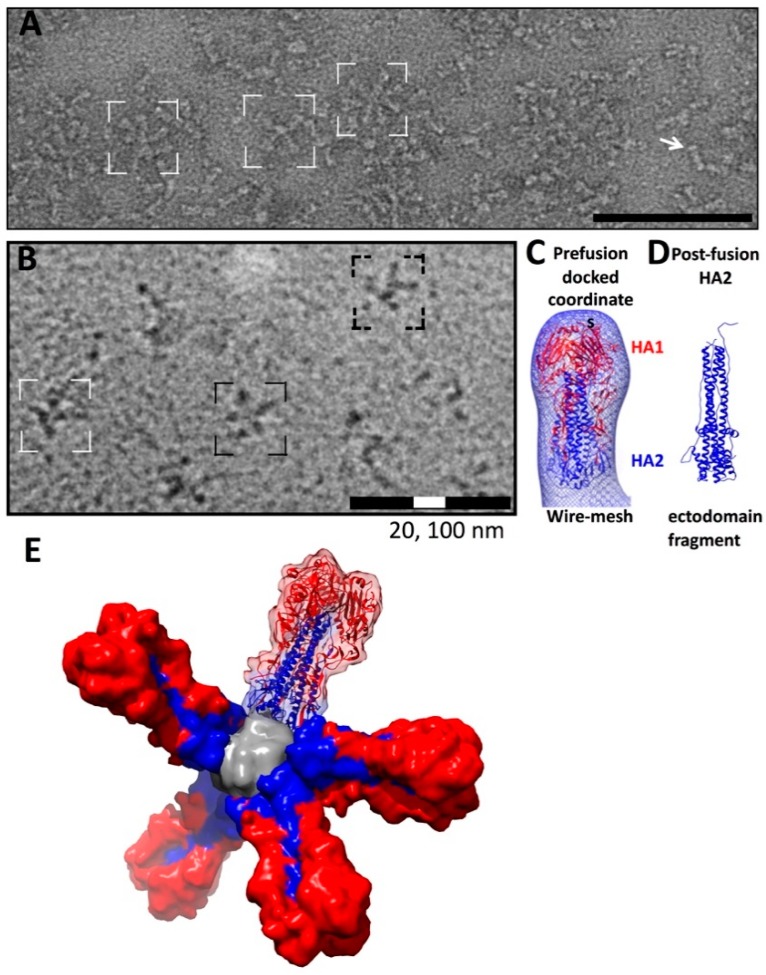
Characterization of HA complexes used as vaccine immunogens by electron microscopy. (**A**) molecular complexes imaged by negative staining EM for a commercial influenza HA subunit-based vaccine for the 2017–2018 influenza season (Fluzone). Density contrast is represented as white. Brackets denote some select HA complexes. An arrow denotes an HA molecule. Scale bar, 100 nm; (**B**) HA complexes of recombinant H7 HA imaged by cryo-electron microscopy. Density contrast is represented as black. Brackets denote some select HA complexes. Scale bars, 100 nm and 20 nm; (**C**) 3D map of H7 HA derived from cryo-EM images of HA complexes. The map is shown as a wire mesh with docked H7 ectodomain coordinates (PDB 4DJ6) in a pre-fusion state and compared with (**D**) post-fusion HA coordinates (PDB 1QU1). HA1 is red with HA2 in blue. (**E**) 3D model of a HA-complex. HA molecules are from coordinates (3LZG) and are shown as molecular surfaces. One constituent HA molecule is shown as a ribbon diagram with a transparent surface. Panels (**A**) and (**E**) are originals and panels (**B**–**D**) were adapted from McCraw et al. [39].

For this review, we imaged one example of a commercial influenza vaccine by negative staining EM (Figure 4A). This is the commercial influenza vaccine Fluzone (high-dose) marketed for the 2017–2018 influenza season. It is a trivalent, inactivated, split vaccine, comprised of HA molecules from influenza type A: H1, H3, and an HA from an influenza B virus. The purified antigens were derived from inactivated influenza virus grown in chicken eggs. Electron microscopy indicated variation in the organization of constituent HA molecules (Figure 4A). HA molecules form micelles or starfish-shaped complexes (Figure 4A, brackets). The starfish shapes result from agglomeration of the hydrophobic transmembrane (TM) domains of different HA trimers after removal of detergent that initially extracted HA trimers from the viral membrane. In a subset of the observed particles, the distinctive peanut-like shape of HA was readily visible (Figure 4A, arrow). The angular arrangement of HA trimers within the starfish-shaped complexes do not conform to a specific rotational symmetry (Figure 4A), thus making the configuration of HA in the vaccine heterogeneous. This differs from a newer class of designed nanoparticles that are symmetric vaccine immunogens that are structurally homogenous and will be discussed later in this review.

Heterogeneity in antigen presentation between different commercially available vaccines is possible, and even likely, as protein composition and production methods are varied. We have previously reported a structural study with recombinant H7 HA by cryo-EM [39], produced in the same proprietary baculovirus-based expression system that is used to produce HA proteins for the commercial vaccine Flublok [37]. This vaccine was approved in the United States in 2013 and uses recombinant protein technology [101]. In contrast to the varied morphologies of HA seen in Fluzone (high-dose), cryo-EM of the recombinant H7 HA revealed complexes with only starfish shapes (Figure 4B). No symmetrical arrangement of constituent HA molecules were observed and the number of constituent HA molecules in the complexes varied from six to eight molecules [39,46]. To determine the conformation of the HA molecules, cryo-EM single particle analysis was carried out by computationally extracting individual HA molecules from the complexes [39]. The HA was in a pre-fusion state as judged by comparing the molecular contour of the 3D map with coordinates for pre-fusion and post-fusion HA ectodomains (Figure 4C,D). We derived 3D molecular models of the HA-complexes by placing C-terminal ends of HA2 molecules toward a central point, which is presumably where transmembrane regions interact to form the complexes [32] (Figure 4E). This starfish model predicts heterogeneity for HA complexes in HA number and arrangement via hydrophobic transmembrane regions at the centroid (Figure 4E). In the model, HA1 heads are on the outside of the starfish. Having HA1 heads projecting out from the starfish may be a structural basis for why the antigenically variable HA1 region could be immunodominant (Figure 4E). While starfish-shape oligomers for vaccines are traditionally made from influenza virus raised in eggs, recombinant HA constructs have been produced in bacteria [95,102,103]. The morphology of the bacterial produced HA starfish was confirmed using negative staining EM, and when combined with antigenicity studies, provided strong evidence that the bacterial product could elicit strain specific antibodies akin to the egg-based production methods [102].

This 3D model for traditional HA subunit influenza vaccines suggests that further engineering of HA to structurally and biochemically improve stem display and immunogenicity could lead to improved immune responses to the conserved stem. Indeed, there are a number of emerging vaccine and design strategies to target the HA stem region [11,14,15,16,26,94,95]. One strategy has been to design chimeric HA proteins with different HA1 heads and conserved HA2 stem regions in order to focus immune responses to the stem region [15,16,94]. Another strategy has been to use structure-guided design of nanoparticles to remove the immunodominant HA1 head and to create a stem displaying nanoparticle immunogen [11]. This strategy is reviewed below.

## 7. Structure-Guided Design of HA-Stem Nanoparticle as Vaccine Immunogen

**Figure 5:**
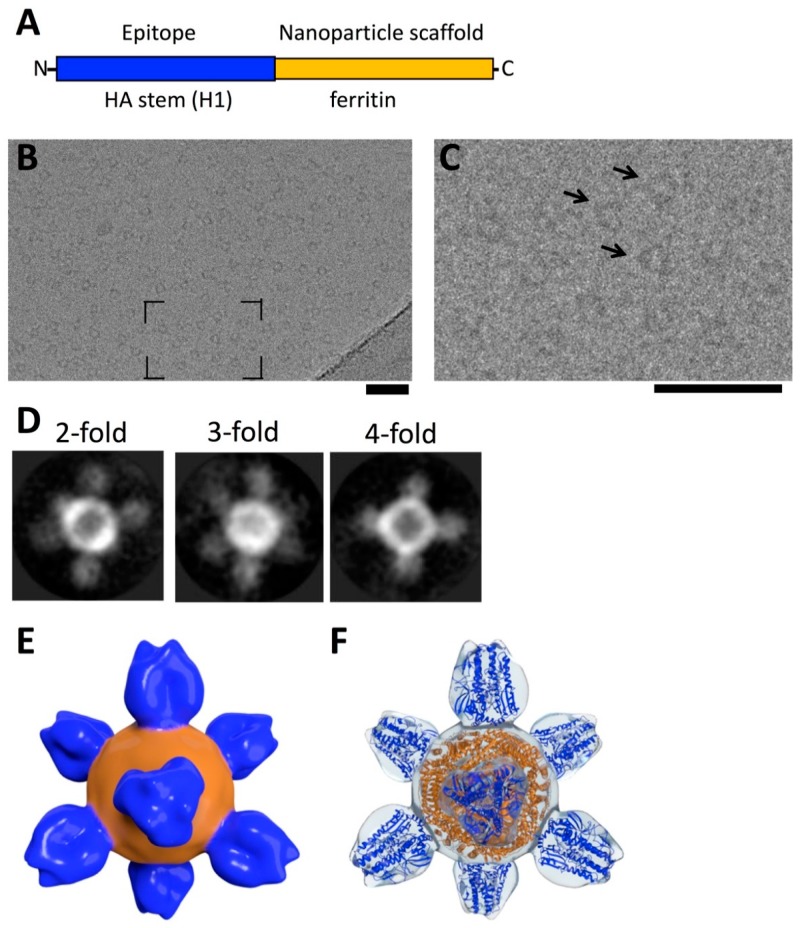
Design concept for the HA-stem nanoparticle and analysis by cryo-electron microscopy. (**A**) schematic organization of an engineered fusion protein made of an epitope fused to a nanoparticle scaffold. In this example the epitope is the conserved stem region of H1 HA (blue) and the nanoparticle scaffold is ferritin (orange); (**B**) cryo-EM of HA-stem nanoparticle. The region that is in brackets is enlarged in panel (**C**); (**C**) HA-stem nanoparticle by cryo-EM with some particles denoted by arrows. Scale bars, 50 nm; (**D**) some examples of reference-free 2D class averages from cryo-EM that display 2-fold, 3-fold, and 4-fold symmetry views consistent with ferritin octahedral symmetry; (**E**) 3D structure of HA-stem nanoparticle solved by single particle cryo-EM. The 3D map is shown as a surface rendering. The map is the deposited map from the 3DEM database (EMD-6332). The regions corresponding to spikes are colored blue and the base orange; (**F**) 3D map of the HA-stem nanoparticle shown as a transparent isosurface with docked coordinates. Coordinates for the designed fusion protein were computationally derived using HA and ferritin sequences [11]. Coordinates corresponding to regions for the HA-stem are in blue and ferritin regions are in orange. The shapes of the HA2-stem regions are consistent with a pre-fusion state of HA2 being displayed on the nanoparticle without the presence of HA1 heads. The original construct is named H1-SS-np and is referred to here as the HA-stem nanoparticle.

Here, we briefly review our cryo-EM contribution and analysis within a larger pipeline for the development of H1-SS-np, an H1 HA stabilized stem construct, displayed on a ferritin-based scaffold (HA-stem) [11]. A structure-guided design approach using tools such as Rosetta [104,105] was employed to predict the structure of a HA-ferritin fusion protein that would display conserved HA-stem epitopes on a ferritin-based nanoparticle scaffold (Figure 5A). The ferritin scaffold assembles from 24 protomers into an approximately spherical entity 10 nm in diameter. The ferritin assembly has octahedral symmetry and the particle has 2-fold, 3-fold, and 4-fold rotational symmetry axes. HA was inserted at the 3-fold rotational symmetry axis, allowing native-like trimers to form on the surface of the ferritin particle. The design features antigen spikes readily accessible to the immune system, yet these same spikes likely hindered formation of crystal contacts and made the particle as a whole refractory to studies by X-ray crystallography.

The HA-stem nanoparticle was readily captured in vitreous ice, where the particles were found to be well dispersed and suitable for image analysis, presenting ring-like structures with small spikes (Figure 5B,C). Cryo-EM was used to determine if the engineered particle retained the ferritin symmetry and epitope display that was fundamental to the design. Initial stages of 3D reconstruction in cryo-EM utilize 2D-class averages, which are model free and make no assumptions about particle symmetry. Averages were found that revealed 2-fold, 3-fold, and 4-fold symmetry axes (Figure 5D), indicating that the HA-stem nanoparticle possessed octahedral symmetry of ferritin. A 3D reconstruction of the nanoparticle resolved the antigenic spikes on a round nanoparticle scaffold (Figure 5E). Hence, cryo-EM played an important role in establishing the structure and epitope conformation for this nanoparticle [11,106].

Because many broadly reactive antibodies to the stem have epitopes only formed in the HA prefusion state [8,17,45,57], it is important to further understand the conformation of the HA2 stem component in the absence of a HA1 head domain. To investigate this, molecular modeling was performed by docking a coordinate model in the cryo-EM map (Figure 5F). This established that the HA2 molecules were in a native-like pre-fusion state on the nanoparticle without the presence of the HA1 head domains. The cryo-EM work provided a structural basis for the observed heterosubtypic protection that the particle imparted against H1N1 and H5N1 influenza virus challenges in animal models [11]. The efficacy of this HA-stem nanoparticle is derived from multiple copies of the pre-fusion state of HA2 being arrayed on the surface of the particle in a symmetrical fashion. This model is consistent with an emerging concept in antigen design in that multivalent displays of viral antigens can increase immunogenicity and efficacy in animal models [10,11,107,108,109].

## 8. Future Efforts in Vaccine Design

Structure-guided immunogen design for infectious diseases is an increasingly viable approach. The use of cryo-EM has been also applied to understand the structure of a number of viral antigens in the context of viral surfaces and vaccine antigens, such as HIV surface glycoproteins [86,87,108,110]. The scientific and commercial viability proof of concept for nanoparticle vaccines has been established for vaccination to hepatitis B virus (HBV) and human papilloma virus (HPV). Both vaccines use nanoparticles constructed from major structural proteins that are the primary antigens on their viral surfaces. The HBV vaccine consists of glycoprotein nanoparticles made from recombinant HBV surface antigen proteins [111,112,113]. For HPV, a vaccine to prevent cervical cancer, the vaccine consists of nanoparticles constructed from the major capsid protein [114,115,116,117,118]. A key development in the HPV vaccine was the observation that expression of the L1 protein created virus-like particles, which were confirmed by electron microscopy to have a surface morphology indistinguishable from native virions [116]. This was foundational in creating a vaccine that has now been shown to be highly efficacious [119,120] and the FDA approved it for use against nine types of HPV [115].

**Figure 6:**
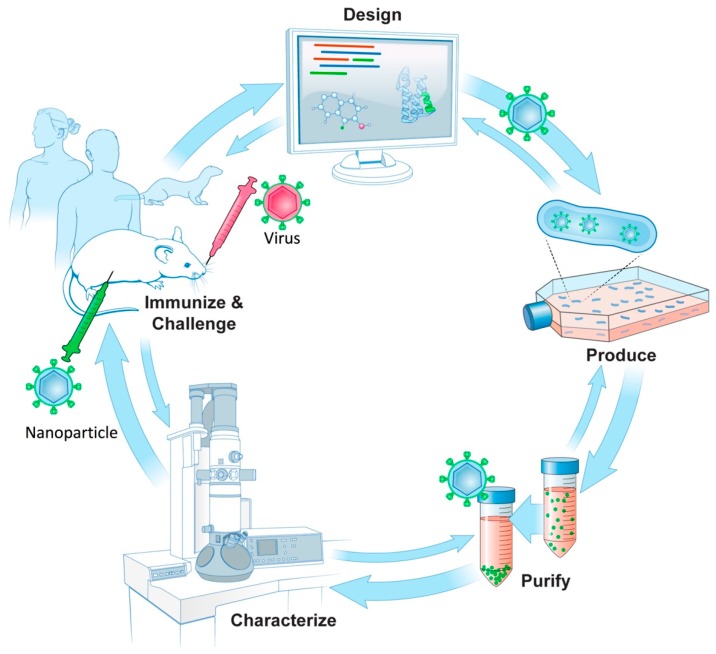
Schematic of structure-guided immunogen design integrated into a vaccine development pipeline. (Design) A conserved epitope from a surface antigen (green) is designed into a fusion protein in order for epitope display on a nanoparticle. Design uses bioinformatics taking into account conserved sequences and epitopes along with structural and molecular modeling of nanoparticle structures in silico. (Produce) To produce nanoparticles, protein encoding DNAs are synthesized and screened for protein expression and nanoparticle formation. (Purify) Nanoparticle production and purification is scaled for further characterization. (Characterize) Immunoassays and biophysical techniques such as electron microscopy are used. 3D structures can be produced by cryo-EM to assess particle integrity, along with epitope display and conformation. (Immunize, Challenge) Immunogenicity and challenge studies using nanoparticles as immunogens can be carried out in animal models, such as mice and ferrets with progression into human studies. Double arrows denote the general iterative nature among steps in the process of structure-guided antigen design within a vaccine pipeline.

Current and future abilities to optimize the design and to model novel immunogens rely heavily on progress in protein modeling and structure prediction [121,122,123]. Particularly representative of these advances are software packages like Rosetta, which can be used to predict and model the structures of designed proteins [104,105]. Structural modeling is emerging as an important computational tool to aid in the design of vaccine nanoparticles [11,109]. The future trend in structure-guided design appears to be an integrated approach that combines computational immunogen design with biochemistry, immunology, vaccinology, systems biology, and structural biology, including X-ray crystallography and cryo-EM [10,11,107,109]. A conceptual workflow for structure-guided design within a vaccine development pipeline is diagrammed in Figure 6. We denote an iterative approach to select stages as icons that represent the design, production and purification of immunogens along with their characterization in conjugation with immunity studies (Figure 6).

In the case of influenza, the structure-guided design of effective HA immunogens coupled with structural determination by X-ray crystallography and cryo-EM indicate a potential paradigm shift in how vaccine immunogen design will be approached in the future [10,11,14,16]. There is an increasing recognition of the power of cryo-EM as a structural technique, as it has aided in the structural determination of influenza epitopes in the context of influenza viruses, designed antigens, immune complexes, chimeric HA molecules, and vaccine nanoparticles [4,11,16,39,45,46,106,124].

Cryo-EM may not have been considered as contributing to vaccine development from a traditional virology or vaccinology perspective. However, from a young investigator’s point of view, the future of vaccine development can be strengthened by strong support in all possible forms to tenure-track investigators, young scientists and trainees who bring expertise from other fields, such as structural biology, biophysics and biochemistry. A paradigm shift appears to be occurring due to some notable successes in structure-guided design such as de novo engineering of icosahedral protein complexes [125]. 

Cryo-EM is currently undergoing rapid innovations on the technical level that would forecast its utility for structural biology. Improved automated data collection and continued improvements in imaging sensitivity by direct-electron detector cameras suggest that the resolution of cryo-EM structures will continue to improve [126,127]. Thus, virus, antigen and vaccine characterization using electron microscopy techniques will likely become an integral part of developing more efficacious influenza vaccines and help to lead to the development of a universal influenza vaccine. Furthermore, the lessons learned from influenza vaccine development can be applied to other pathogens, leading to a reduction in the morbidity and mortality associated with current and emerging infectious diseases worldwide.
